# Case report: A rare case of simultaneous necrotizing fasciitis of the breast and forearm

**DOI:** 10.3389/fmed.2024.1413593

**Published:** 2024-06-14

**Authors:** Ruofei Xu, Tingting Fang, Weikang Cai

**Affiliations:** ^1^Orthopedics Department, Longyou County People's Hospital, Longyou, China; ^2^Dermatology Department, Longyou County People's Hospital, Longyou, China; ^3^Orthopedics Department, Yongkang Hospital of Traditional Chinese Medicine, Yongkang, China

**Keywords:** necrotizing fasciitis, severe implications, early surgical treatment, diabetes mellitus, hepatitis B

## Abstract

Necrotizing fasciitis is an aggressive bacterial infection that causes necrosis of the fascia and subcutaneous tissues with rapid progression and high mortality. Early stages often lead to misdiagnosis, resulting in improper treatment and severe implications. This case study presents a patient with diabetes mellitus combined with hepatitis B who rapidly developed necrotizing fasciitis of the left forearm and left breast after trauma and controlled the infection with early surgical treatment. It is worth noting that early surgical exploration is the gold standard for the diagnosis of necrotizing fasciitis and is the most effective means of reducing mortality and amputation rates in necrotizing fasciitis.

## 1 Introduction

Necrotizing fasciitis (NF) is a rapidly progressive skin infection characterized by necrosis of fascia and subcutaneous tissue (1). The incidence of necrotizing fasciitis is low, estimated at 0.86 to 32.64 cases per 100,000 population (2). Due to the insidious onset and rapid progression of necrotizing fasciitis, the disease is easily misdiagnosed in its early stages, and if left untreated, the resultant disability and mortality rates are extremely high (3). This report documents a relatively rare case of necrotizing fasciitis occurring simultaneously in the left forearm and breast, which was treated aggressively and recovered well.

## 2 Case presentation

A 46-year-old male (height: 175 cm, weight: 76 kg) presented with a half-month history of left forearm swelling and pain with extremity numbness. The patient was injured by a heavy object on his left forearm half a month ago. It was not taken seriously and not treated at that time, and then the swelling and pain in the forearm became more and more severe, so he came to our hospital for treatment. At that time, the patient had a 7-year history of diabetes mellitus, which was treated with subcutaneous insulin for 3 months after the onset of the disease, and then switched to oral hypoglycemic medication, which was discontinued on its own 3 years ago, and his blood glucose was not monitored again. There was a history of hepatitis B. There was no history of surgery, no history of food or allergies, and no relevant personal or family history.

**Figure 1:**
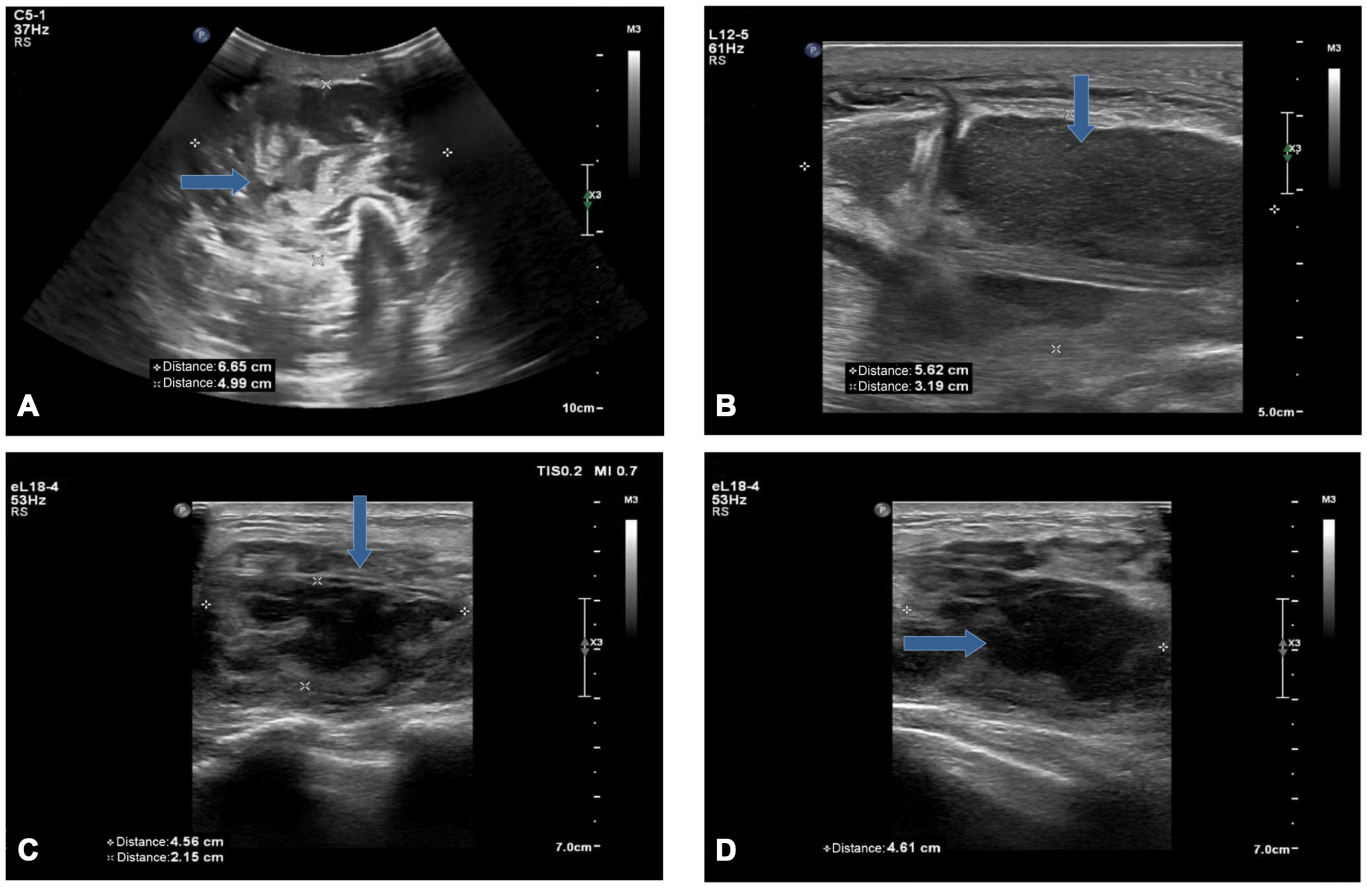
Ultrasound image of the left forearm **(A,B)** and left breast **(C,D)**: A mixed echogenic mass was seen in the deep fascial and muscular spaces **(A)**, with irregular dark areas inside **(B)**. An inhomogeneous hypoechoic area **(C)** in the breast with poorly defined borders containing an irregular dark area **(D)** (blue arrow).

**Figure 2:**
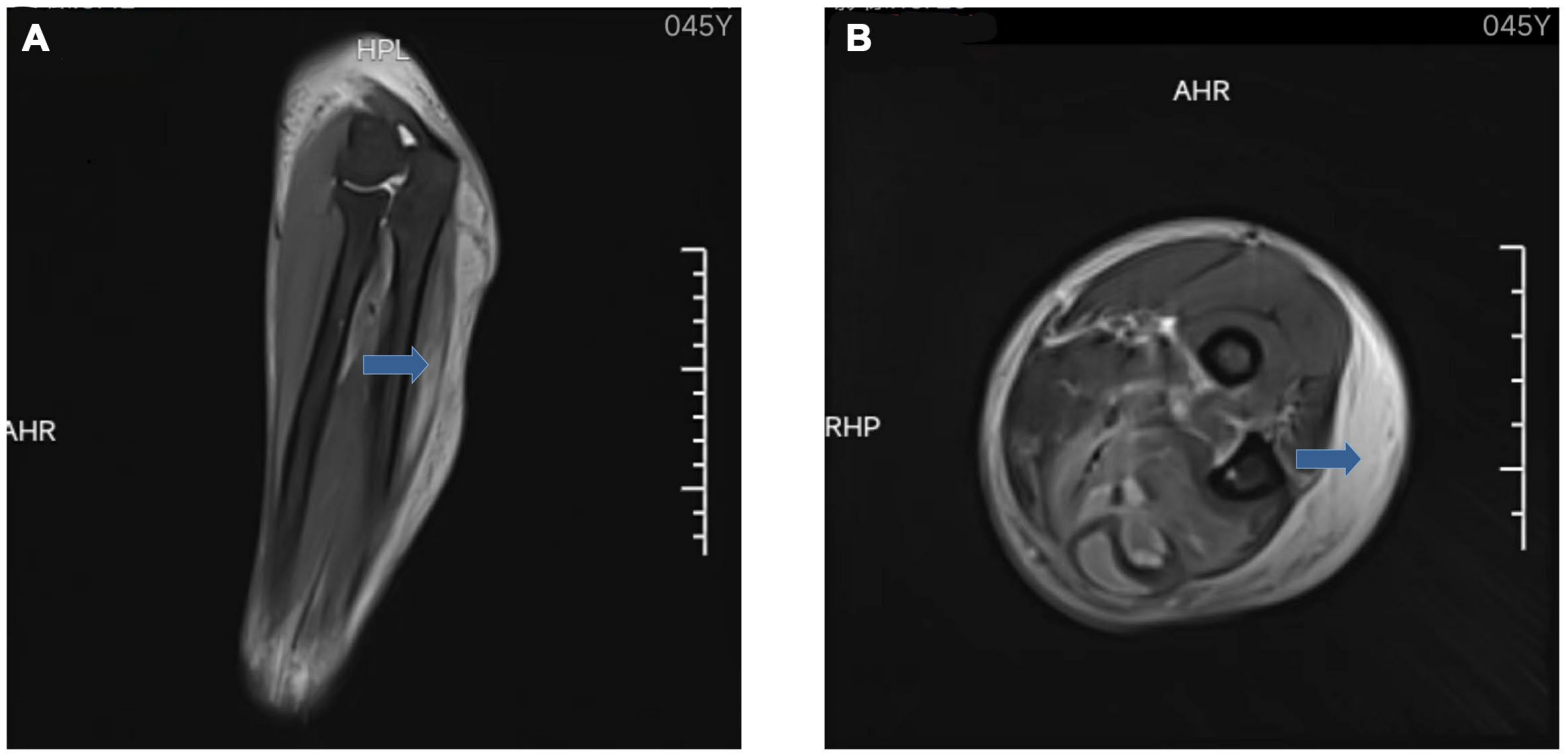
MRI of the left forearm: coronal **(A)** and axial **(B)** showed extensive tissue inflammatory changes within the deep fascia (blue arrow).

**Figure 3:**
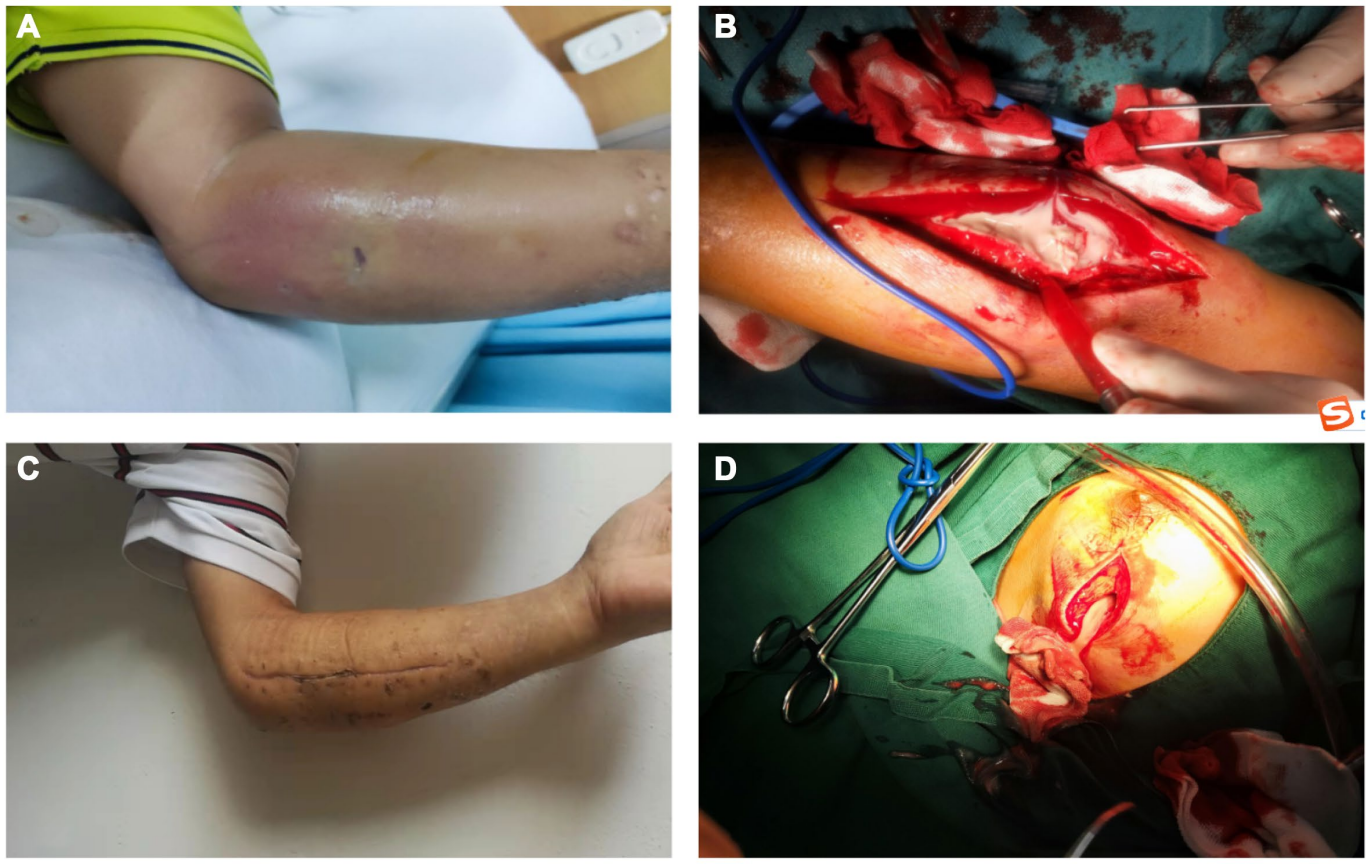
**(A)** On the 3rd day of hospitalization, the patient’s skin on the left forearm was markedly red and swollen. **(B)** In the debridement of the left forearm, we found a large amount of pus under the fascia. **(C)** On the 14th day after the second surgery, we removed the stitches from the patient and found that the wounds were healing well. **(D)** During surgery, a huge abscess cavity was found in the left breast, containing a large amount of milky pus.

The outpatient doctor found that his blood sugar was high (fasting blood glucose: 11.96 mmol/L) and accompanied by dry mouth, polydipsia, polyuria, and he was hospitalized in the endocrinology department, where he was diagnosed with type 2 diabetes mellitus and soft tissue injury. Ultrasound image of the left forearm showed a mixed echogenic mass in the deep fascial and intermuscular spaces with an irregular dark area inside (Figures 1A,B). Magnetic resonance imaging (MRI) of the left forearm showed extensive tissue inflammatory changes within the deep fascia (Figure 2). Computed tomography (CT) of the chest did not show any positive results. The internist gave him subcutaneous insulin for hyperglycemia and intravenous cefuroxime against infection. After 3 days of treatment, the swelling of the left forearm was found to be more severe, with elevated skin temperature and obvious pressure pain (Figure 3A). He was then referred to orthopedics.

On the day of transfer to the orthopedic department, we were surprised to find a lump in the patient’s left breast that was painful when pressed, and an ultrasound that showed heterogeneous hypoechoicity and dark areas in the left breast (Figures 1C,D). We reviewed the patient’s post-hospitalization laboratory findings (Table 1) and imaging evaluation and combined them with the Laboratory Risk Indicator for Necrotizing Fasciitis (LRINEC) score. We considered this case to be most likely necrotizing fasciitis, with the patient’s LRINEC score being 6 (total score: 13). Considering the rapid spread of the necrotic area and the presence of septic shock (blood pressure: 85/55 mmHg, heart rate: 104 beats/min, respiratory rate: 24 breaths/min, temperature: 38.5°C), we performed an emergency surgical intervention under general anesthesia, in which we found a large amount of grayish-white pus coming out of the subcutaneous tissues and fascial layers of the left forearm, with “cotton-wool” changes, a positive “finger test.” There was a large abscess cavity in the left breast which contained a large amount of milky pus. We incised the abscess cavities, removed the necrotic tissues, and cleaned the wounds with large amounts of saline, 3% hydrogen peroxide, and povidone-iodine solution (Figures 3B,D). Vacuum-sealed drainage (VSD) was then applied in the left forearm fascial space and left breast pus cavity.

**Table 1:** Laboratory risk indicator for necrotizing fasciitis (LRINEC) score.

Variable (units)	Value	Reference value	LRINEC score
White blood cell (10^9^/L)	27.0	4.0–10.0	2
Hemoglobin (g/L)	126	120–160	1
Sodium (mmol/L)	133.5	135.0–145.0	2
Glucose (mmol/L)	11.53	3.61–6.11	1
Creatinine (μmol/L)	62.0	59.0–104.0	0
C-reactive protein (mg/L)	115.17	0.0–10.0	0

On the 7th day after surgery, we removed the VSD devices from the left forearm and left breast and found that the wounds had a good supply of fresh blood with no visible pus, and then excised the necrotic tissues, rinsed the wounds, sutured the skin and covered the VSD devices. We performed separate cultures of the pus from the left forearm and left breast from the first surgery, both of which showed positive results for *Staphylococcus aureus*. Drug susceptibility testing showed it to be resistant to penicillin and susceptible to erythromycin, levofloxacin, clindamycin, vancomycin, cephalexin and moxifloxacin. Therefore, we gave the patient 10 consecutive days of clindamycin and levofloxacin to treat the inflammation (The day before stopping the medication, laboratory tests revealed that the patient’s white blood cell count was 8.2 × 10^9^/L, C-reactive protein was 15 mg/L). On the 7th day after the second surgery, we removed the VSD devices and observed that the patient’s condition was stable with good wound healing and he was discharged from the hospital. On the 14th day after the second surgery, we removed the patient’s sutures (Figure 3C).

## 3 Discussion

Necrotizing fasciitis may involve soft tissues throughout the body, most commonly the abdomen, perineum, buttocks, and extremities (4). In this study, the patient was first found to have an infection in the left forearm and later in the left breast, which was considered to be a possible hematogenous infection. The primary cause of disease flare-ups may be tissue infections caused by trauma, while diabetes and chronic hepatitis may also be the predisposing causes of the disease. The low prevalence of NF and the low specificity of the early clinical manifestations are highly susceptible to misdiagnosis. Timely and accurate diagnosis is the key to perform debridement surgery at later stage and reduce the morbidity and mortality.

Previous studies have found that the Laboratory Risk Indicator for Necrotizing Fasciitis (LRINEC), which includes C-reactive protein, white blood cell count, hemoglobin, serum sodium, serum creatinine, and serum glucose laboratory markers, is important for the early diagnosis of NF and the differential diagnosis from other soft-tissue infections, including severe cellulitis and abscesses. With a total LRINEC score of 0 to 13, a score of ≥6 suspects NF with a probability of 50 to 75%, suggesting an intermediate risk, whereas a score of ≥8 strongly predicts NF with a probability of more than 75%, suggesting a high risk (5–7). Despite several published data showing good clinical correlation of LRINEC scores, a negative LRINEC score must not be considered a reliable indication to exclude a diagnosis if NF is clinically suspected (8, 9).

Imaging is a powerful adjunct to early diagnosis. US and MRI have high sensitivity and specificity in the diagnosis of NF (10). The likelihood of NF in patients with clinically suspected NF is higher when US examination reveals the presence of fluid accumulation along the deep fascia. The diagnostic accuracy of NF was 72.7% when US examination revealed fluid accumulation at a depth of more than 2 mm in the affected area. For prognostic prediction of NF, the presence of fluid accumulation along the deep fascia may indicate a longer hospital stay and a risk of amputation or death in patients with NF (11). MRI is the gold standard for the diagnosis of soft-tissue infections, with excellent soft-tissue contrast resolution, and has a sensitivity of 93.0% in the diagnosis of NF (12, 13). Thickening of deep fascia and multicompartmental involvement in the MRI examination is a typical feature of NF, which can be more accurately indicative of NF (14).

Imaging, laboratory tests, and the LRINEC scoring system are important aids for early diagnosis, but the most reliable method of confirming the diagnosis remains surgical exploration. Early and adequate surgical debridement is the key to treating NF. Numerous studies have shown that delayed surgical intervention and inadequate initial debridement result in significantly higher morbidity and mortality (15). In this case, we achieved a satisfactory outcome with prompt debridement and postoperative use of VSD devices, coupled with the judicious use of antimicrobial drugs and other therapies.

In recent years, research on the diagnosis and treatment of NF has made some achievements, but the mortality and amputation rates of NF have not been significantly controlled (16). This is mainly due to the rapid progression of NF, the difficulty of early recognition, and the lack of clear diagnostic criteria, which are the focus of future research on NF.

Future research efforts will need to monitor ongoing changes in microbiologic etiology and enhance imaging and diagnostic technologies to improve the ability to detect and treat disease at an early stage. To date, there are few large prospective studies comparing imaging and diagnostic modalities. In addition, further clinical studies are needed to determine which current or future complementary therapies may additionally affect patient prognosis and survival time.

## 4 Conclusion

In our case, necrotizing fasciitis occurring in the ipsilateral arm and breast, potentially resulting from hematogenous spread, is a rare condition that has not been reported before. The VSD device provides a uniform negative pressure on the wound, which can continuously and effectively remove necrotic tissue and exudate from the fascial space and prevent the spread of infection. We believe that early surgical treatment is important for necrotizing fasciitis, and also the postoperative use of VSD devices plays a key role, which can be used as a reference for the treatment of necrotizing fasciitis.

## Data availability statement

The original contributions presented in the study are included in the article/supplementary material, further inquiries can be directed to the corresponding author.

## Ethics statement

The studies involving humans were approved by Ethics Committee of Longyou People’s Hospital. The studies were conducted in accordance with the local legislation and institutional requirements. The participants provided their written informed consent to participate in this study. Written informed consent was obtained from the individual (s) for the publication of any potentially identifiable images or data included in this article.

## Author contributions

RX: Conceptualization, Data curation, Writing – original draft. TF: Data curation, Visualization, Writing – review & editing. WC: Conceptualization, Methodology, Writing – original draft.
